# All You Need to Know About TACE: A Comprehensive Review of Indications, Techniques, Efficacy, Limits, and Technical Advancement

**DOI:** 10.3390/jcm14020314

**Published:** 2025-01-07

**Authors:** Carolina Lanza, Velio Ascenti, Gaetano Valerio Amato, Giuseppe Pellegrino, Sonia Triggiani, Jacopo Tintori, Cristina Intrieri, Salvatore Alessio Angileri, Pierpaolo Biondetti, Serena Carriero, Pierluca Torcia, Anna Maria Ierardi, Gianpaolo Carrafiello

**Affiliations:** 1 Department of Diagnostic and Interventional Radiology, Foundation IRCCS Cà Granda—Ospedale Maggiore Policlinico, Via Francesco Sforza 35, 20122 Milan, Italy; carolinalanza92@gmail.com (C.L.); pierpaolo.biondetti@gmail.com (P.B.); serena.carriero@gmail.com (S.C.); pierluca.torcia@policlinico.mi.it (P.T.); annamaria.ierardi@policlinico.mi.it (A.M.I.); gianpaolo.carrafiellol@unimi.it (G.C.); 2 Postgraduate School in Radiodiagnostics, Università degli Studi di Milano, 20122 Milan, Italy; velio.ascenti@unimi.it (V.A.); gaetano.amato@unimi.it (G.V.A.); giuseppe.pellegrino@unimi.it (G.P.); sonia.triggiani@unimi.it (S.T.); jacopo.tintori@unimi.it (J.T.); 3 Postgraduate School in Diangostic Imaging, Università degli Studi di Siena, 20122 Milan, Italy; cristinaintrieri95@gmail.com; 4 Faculty of Health Science, Università degli Studi di Milano, Via Festa del Perdono 7, 20122 Milan, Italy

**Keywords:** transarterial chemoembolization, liver, hepatocellular carcinoma

## Abstract

Transcatheter arterial chemoembolization (TACE) is a proven and widely accepted treatment option for hepatocellular carcinoma and it is recommended as first-line non-curative therapy for BCLC B/intermediate HCC (preserved liver function, multifocal, no cancer-related symptoms) in patients without vascular involvement. Different types of TACE are available nowadays, including TAE, c-TACE, DEB-TACE, and DSM-TACE, but at present there is insufficient evidence to recommend one TACE technique over another and the choice is left to the operator. This review then aims to provide a comprehensive overview of the current literature on indications, types of procedures, safety, and efficacy of different TACE treatments.

## 1. Introduction

Hepatocellular carcinoma (HCC) is the third leading cause of cancer-related deaths worldwide and one of the most common causes of death among patients with cirrhosis [1]. The annual incidence of HCC in the cirrhotic population is 3–5% [2]; therefore, diagnostic surveillance in this category of high-risk patients is crucial to diagnose the tumors in early stages. Ultrasound (US) is the most widely used imaging test for surveillance because of its low costs and non-invasive nature, with a sensitivity of 60–80% in experienced hands [3]. Different new technologies have been introduced to maximize the effective role of US, such as shear-wave elastography (SWE) with the possibility to measure the stiffness of nodules and contrast-enhanced US (CEUS) is also used nowadays as a standardized classification system, the CEUS Liver Imaging Reporting and Data System (CEUS LI-RADS) [4,5,6]. Treatment approaches for HCC can be classified into three categories: potentially curative, palliative, and symptomatic. Hepatic resection, transplantation, and image-guided locoregional treatments, which include thermal ablation (radiofrequency or microwave), transarterial chemoembolization (TACE), and radioembolization, represent potentially curative treatment.

Despite surveillance, a large number of patients receive a diagnosis when they are no longer responsive to curative treatments (such as liver transplantation, surgical resection, and ablation). In addition, the high expense and lack of organ donors restrict the use of liver transplantation, which increases the need for innovative HCC treatment approaches [7].

TACE is widely acknowledged for its efficacy and as a valuable treatment option in managing HCC.

Based on the Barcelona Clinical Liver Cancer (BCLC) system and endorsed by the American Association for the Study of Liver Diseases (AALSD) and by the European Association for the Study of the Liver (EASL) guidelines for HCC management, TACE is recommended as primary therapy for intermediate-stage HCC (BCLC-B), defined as a large multinodular cancer associated with Child–Pugh stage A–B and Eastern Cooperative Oncology Group performance status (PS) of 0 [8,9,10]. Nevertheless, TACE is often performed in the Asia-Pacific region, even for patients with macrovascular invasion (with no extrahepatic metastasis), and according to some literature reports, it may grant survival benefits when conducted in selected patients with good liver function [11,12]. Moreover, TACE can be employed in BCLC-0 or A/early HCC in cases where resection or ablation is contraindicated or as a bridge to transplantation [13].

Various types of TACE are available, including transarterial embolization (TAE), conventional TACE (c-TACE), drug-eluting microspheres TACE (DEM-TACE), and degradable starch microspheres TACE (DSM-TACE), but at present there is insufficient evidence to recommend one TACE technique over another and the choice is left to the operator [14].

Several different types of DEM exist, and four are approved for TACE, including DC Bead, HepaSphere, TANDEM, and LifePearl.

Lipiodol for c-TACE or drug-eluting beads for DEB-TACE as embolic agents are the most commonly used treatments. Degradable starch microspheres (DSMs) have only recently emerged as an alternative. The most relevant difference between c-TACE and DSM-TACE is transient vessel occlusion with a half-life time of approximately 40 min for DSM-TACE compared to the prolonged washout of Lipiodol (5–12 weeks) in c-TACE [15].

The role of hepatic arterial infusion chemotherapy (HAIC) in the treatment of hepatocellular carcinoma (HCC) will also be examined, along with other innovative techniques, such as balloon-assisted transarterial chemoembolization (b-TACE) (Table 1).

New frontiers, such as the application of combined techniques and the potential of artificial intelligence, will also be assessed.

This review then aims to provide a comprehensive overview of the current literature on indications, types of procedures, safety, and efficacy of different TACE treatments.

## 2. Indications

According to the Barcelona Clinical Liver Cancer (BCLC) system, TACE is indicated as first line-treatment for intermediate (BCLC-B) patients associated with Child–Pugh stage A–B and Eastern Cooperative Oncology Group performance status (PS) of 0 and as an alternative to other not practicable or failed curative treatments (surgical resection, local ablation, systemic therapies) in very early (BCLC-0) and early (BCLC-A) patients [16,17,18].

Using five clinical indicators of liver disease, including total bilirubin level, serum albumin level, prothrombin activity, ascites (none/mild/moderate-severe), and hepatic encephalopathy (none/grade I, II/grade III, IV), the Child–Pugh score is used to assess liver function. The diagnosis of HCC is based on imaging (US/CESU/CT/MRI) and serum tumor makers such as alpha-fetoprotein (AFP) and des-γ-carboxy prothrombin (DCP).

The underlying disease’s impact on everyday living abilities is measured by the performance status (PS) scale.

Hence, the possible aims of TACE are the follows: (a) reducing the total tumor burden to within the transplant criteria (downstaging), (b) controlling tumor growth in a patient who is on the transplant list (bridging), and (c) increasing survival in patients not eligible for transplantation (palliative) [19].

TACE is often used as a bridge therapy before liver transplantation (LT) when the expected waiting period for an organ is more than 6 months, and it has been associated with a lower dropout rate for patients awaiting LT.

TACE is indicated for BCLC-B stage patients who have massive or multinodular tumors but no macrovascular invasion or extrahepatic metastases. TACE can function as a downstaging treatment in patients with BCLC-B stage to achieve the threshold of surgical resection or LT [20].

Absolute and relative contraindications for TACE include extensive bilobar tumor load, decompensated liver disease, untreated large varices, large tumor diameter, severe comorbidities, and compromised portal vein integrity due to thrombosis or hepatofugal flow [21,22,23]. Due to the increased risk of hepatic ischemia and failure, portal vein thrombosis is still considered a relative contraindication to TACE; anyway, some research revealed that embolization of hepatic artery branches might be conducted successfully even in these settings [24].

According to the BCLC staging system, advanced HCC with vascular invasion or extrahepatic metastasis is considered a contraindication for TACE.

Some conditions are correlated with failure of treatment for HCC. In fact, the Asia-Pacific Primary Liver Cancer Expert (APPLE) meeting proposed the concept of TACE unsuitability, defining the treatment for HCC as follows: (i) unlikely to respond to TACE in case of confluent multinodular type, massive or infiltrative type, simple nodular type with extranodular growth, poorly differentiated type, intrahepatic multiple disseminated nodules, or sarcomatous changes after TACE; (ii) likely to develop TACE failure/refractoriness in case of up to seven criteria out nodules; and (iii) likely to become Child–Pugh B or C class after TACE in case of up to seven criteria out nodules (especially, bilobar multifocal HCC) or modified albumin bilirubin (ALBI) grade 2b.

The “up to seven” criteria is an expanded guideline for selecting candidates for liver transplantation in hepatocellular carcinoma (HCC) cases, introduced as an alternative to the Milan criteria while maintaining good survival outcomes. The criteria allow for the sum of the largest tumor’s size (in centimeters) and the total number of tumors to be up to seven. Modified albumin-bilirubin (ALBI) Grade 2b is a sub-classification within the ALBI system used to evaluate liver function and differentiate Grade 2 in two subclassifications, with Grade 2b indicating patients with moderate liver dysfunction who are still eligible for certain treatments, like locoregional therapies or systemic therapies, but may have a higher risk of complications compared to those with Grade 2a [25].

In clinical practice, decision making is most challenging for patients who do not have clear contraindications but also do not perfectly match the ideal TACE candidate profile (ECOG 0, low tumor burden, well-preserved liver function). Although a better prognosis does not always equate to treatment benefit, it is crucial to use tools that refine the decision-making process for the first TACE treatment and identify patients at risk of TACE-related harm [23,26].

## 3. Procedural Equipment and Preparation

As for the patient preparation, a fast of 4–6 h is recommended before the procedure and peripheral venous access and endorsed informed consent are mandatory. Moreover, monitoring of vital parameters and completion of the pre-procedural safety checklist are strongly suggested [27].

### 3.1. Vascular Access

It is possible to perform vascular access at the radial (RA) or femoral (FA) artery using the Seldinger technique. As evidenced by a recent meta-analysis, the success rate of femoral access (100%) was slightly higher than that of radial access (99.1%) [28]. By contrast, a retrospective study conducted by You et al. (2023) suggested that transradial access may offer certain advantages [29]. According to mRECIST evaluations, patients in the RA group exhibited a higher partial response rate, a lower incidence of hepatic arterial spasm, and a lower progression rate compared to patients in the FA group. Nevertheless, it is also essential to consider that, even if the radial artery is confirmed to be patent at ultrasound examination, it may still be preferable to avoid radial access in certain cases. Such circumstances may arise in cases where the patient requires dialysis or if there is a negative Allen test, severe peripheral vascular disease, or a prior history of severe vascular tortuosity or obstruction. It would be prudent to refrain from using the FA as the access point in elderly or obese patients, as well as in those with coagulopathy due to hepatic cirrhosis, given the elevated risk of bleeding complications associated with femoral arterial catheterization in these individuals. There appears to be a discrepancy in the literature regarding the procedural time for radial access for TACE. While some authors, such as Ghosh et al. [30], have reported longer procedural times for the radial access site, others have found similar times between the two access sites [29,31] or even lower times for the transradial approach [32], as observed by Wu et al. In conclusion, there is currently no conclusive scientific data supporting the use of radial over femoral artery access. In clinical practice, both strategies are employed, and the decision is often influenced by procedural considerations, patient anatomy, and operator preference. Femoral access is still more frequently used because of its well-established role in interventional radiology, even though radial access is likely correlated with a decreased incidence of vascular complications and improved patient comfort. However further research, including randomized controlled trials, is needed to determine whether one approach offers clear advantages in terms of procedural success, safety, or clinical outcomes in TACE.

As far as the technical aspects of transradial vascular access, after local anesthesia, the left radial artery is punctured with a 21-G needle and then a 4F or 5F vascular introducer sheath is inserted over a 0.021-inch microwire. To avoid vasospasm and to decrease the risk of clot formation, a cocktail mix of 2000 IU oh heparin, 2.5 mg of verapamil, and 2 mL of 2% Lidocaine is injected through the sheath. For the transfemoral approach, the puncture is performed with a 18G needle and a 5F vascular sheath is introduced over a 0.021-inch guidewire.

**Figure 1:**
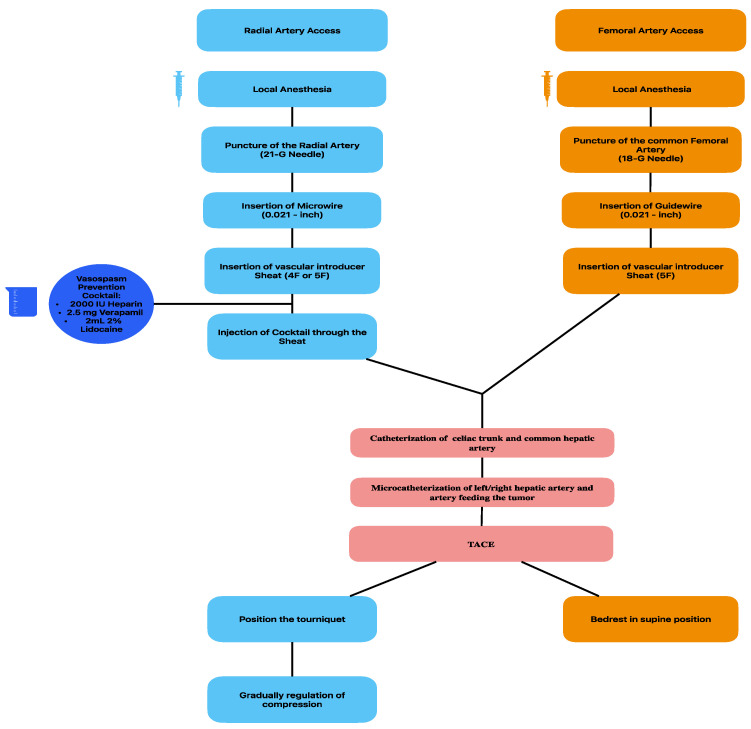
Flow chart illustrating the procedural steps for radial and femoral arterial access in trans arterial chemoembolization (TACE), including patient preparation, access site selection, catheterization, and post-procedure care.

The following passages are similar for both vascular accesses: a diagnostic catheter is advanced into the abdominal aorta and then navigated to the celiac trunk and/or superior mesenteric artery, depending on the target vessel for embolization. Super-selective catheterization is then performed using a microcatheter, which is pushed into the hepatic artery feeding the tumor (Figure 1).

After TACE is performed, FA access requires bed rest in the supine position for at least 6 h if a closure device is used for hemostasis and for 10 h if manual compress is preferred.

The choice between the two accesses is based on the patient and IR decision. As reported in the literature, patients preferred RA access due to less periprocedural pain and shorter hospitalization time [31,33,34].

### 3.2. Guidance Systems

TACE is typically conducted using planar two-dimensional digital subtraction angiography (DSA), often necessitating additional oblique views and contrast medium injections to accurately visualize the feeding vessels supplying the targeted tumor [35]. Recently, technical advancements have enabled the reconstruction of three-dimensional (3D) rotational C-arm computed tomographic (CT) scans using soft tissue windows, creating CT-like images with a flat-panel DSA unit. Focusing on TACE, this combination of vascular and soft tissue visualization offers potential benefits as it allows a clear identification of the relationship between the targeted tumor and its arterial supply [35].

Technological developments have also led to the integration of cone-beam CT (CBCT) on the C-arm of the angiographic machine, and this advancement has been demonstrated to have more tumor and tumor feeder detection in comparison to DSA [36,37,38].

CBCT typically involves higher radiation doses than digital subtraction angiography, and multiple CBCT scans may lead to patient overexposure; however, offering 3D anatomical information, a single CBCT scan can replace multiple digital subtraction angiographies [18]. CBCT, providing intraprocedural three-dimensional volumetric imaging, is superior to standard two-dimensional angiography in the detection of HCC, allowing an improvement in the visualization and targeting of tumors and all their feeding arteries. Numerous studies on phantoms, animals, patients, and medical staff have examined the X-ray exposure linked to CBCT. Depending on factors like tube voltage, tube current, filter thickness and material, and number of projections, the X-ray exposure may differ between manufacturers. For a single abdominal CBCT scan, the patient’s estimated effective dose is between 3 and 10 mSv. The additional use of CBCT decreases the deterministic risk (cumulative dose) when compared with a procedure using fluoroscopy and DSA alone and facilitates the procedure, potentially reducing the procedure time [39]. Additionally, safe and effective TACE with CBCT can decrease the number of future TACE sessions, ultimately reducing overall radiation exposure to patients [18,38]. Since contrast medium is directly injected into the hepatic artery for CBCT scanning, it produces pure hepatic arterial phase images similar to those of a CT hepatic arteriography, permitting precise visualization of fine hepatic arteries and providing high sensitivity for detecting hypervascular tumors [18]. Moreover, the development of dual-phase CBCT has led to major tumor detection compared to single-phase CBCT alone [36]. Although recent advances have introduced motion artifact correction software, CBCT is prone to motion artifacts caused by cardiac and respiratory movements and cooperative respiratory motion control remains essential for acquiring high-quality CBCT images; therefore, it is crucial to exercise caution when interpreting significantly degraded CBCT images [18]. CBCT plays a crucial role in detecting occult nodules of HCCs and fine tumor-feeding arteries, navigating a microcatheter to the target feeding arteries, identifying extrahepatic collateral supply, preventing non-target embolization, and assessing the completeness of chemoembolization treatment during the procedure [18,40]. Checking the embolized area with intraprocedural CBCT has been demonstrated to decrease local recurrence [41,42].

All these demonstrated advantages in the correct use of CBCT have led international guidelines to actively integrate it during TACE treatments [12,37,40].

A novel approach for CBCT consists of TACE guidance software, such as automated tumor feeder detection (AFD), which can increase the accuracy of treatment in the detection of tumor feeders compared to DSA.

The essential process includes 3D segmentation of the targeted lesion(s) on the CBCT dataset and automatic extraction of candidate feeders, starting from the micro-catheter tip and extending to the targets [36].

### 3.3. Chemodrugs

The ideal agent for use in TACE remains undetermined, and there is currently no established biomarker to guide the selection of the most appropriate therapeutic agents for TACE. This makes it difficult to select the most effective TACE agents for patients with hepatocellular carcinoma (HCC) at different stages. As a result, the choice of treatment is influenced by multiple factors, including the cancer’s stage, the patient’s overall health status, the presence of additional medical conditions, and the specific tumor characteristics.

Numerous studies over the years have compared the effectiveness of the most frequently used chemotherapy drugs in TACE, such as Doxorubicin (Adriamycin), Epirubicin, Cisplatin, Mitomycin C, and Miriplatin [43]. These drugs work through different mechanisms and exhibit varying degrees of efficacy against HCC, allowing for their use either individually or in combination. Combining multiple drugs with embolization may enhance their effectiveness, reduce recurrence rates, and improve survival outcomes [44]. Among these agents, anthracyclines, including Doxorubicin and Epirubicin, are the most commonly used in TACE for HCC patients [40]. Various studies have investigated the effectiveness of single-agent TACE, yielding inconsistent results. One prospective study found no significant difference in adverse events or overall survival between Cisplatin-Lipiodol suspension and Epirubicin-Lipiodol emulsion in patients with recurrent HCC [45], whereas retrospective studies suggested a more favorable response to Cisplatin compared to Doxorubicin or Epirubicin [45,46]. The availability of Miriplatin is confined to Japan; hence, the literature is limited about its efficacy and shows no better therapeutic efficacy compared to Epirubicin [47,48].

One of the most common chemotherapy drugs used for TACE is Doxorubicin. In the case of c-TACE, Doxorubicin doses generally range between 30 and 75 mg/m^2^, with a maximum permissible dose of 150 mg. This drug is typically combined with 5 to 20 mL of Lipiodol. For DEB-TACE, the chemotherapy dosage depends on the stage of the disease. Patients with limited disease—defined by the Milan criteria for liver transplantation as a single tumor ≤ 5 cm, or multiple tumors (up to three, each ≤ 3 cm)—should receive 50 to 75 mg of Doxorubicin per treatment. This amount is loaded into one vial containing 2 mL of DC Beads, corresponding to a loading dose of 25 to 37.5 mg of Doxorubicin per mL of beads. For patients with more advanced disease, the recommended dosage per treatment increases to a maximum of 150 mg of Doxorubicin, loaded into two vials of DC Beads [18].

For DSM-TACE in HCC management, the manufacturer’s standard protocol involves gradually injecting the first 4 mL of EmboCept (450 mg/7.5 mL) mixed with 6 mL of non-ionic contrast medium. This is combined with Doxorubicin at a dose of 50 mg/m^2^, adjusted according to body surface area. However, many centers prefer administering a fixed dose of 50 mg of Doxorubicin—lower than the prescribed amount—to reduce systemic toxicity. This mixture is diluted in 5–10 mL of saline solution. The remaining 3.5 mL of EmboCept, combined with an equivalent amount of contrast medium, is subsequently injected until arterial flow stasis is achieved [49] (Table 2).

Drug selection for TACE in HCC patients is also based on previous treatments, tumor features, patient comorbidities, and the neutrophil/lymphocyte ratio (NLR). A recent meta-analysis investigated the role of NLR as a systemic immune marker of patients affected by HCC [50]. The rationale is linked to the role of inflammatory factors in cancer development and the NLR is a systemic immune marker that reflects the body’s level of inflammation. Wang et al. concluded that the NLR is linked to the overall survival of HCC patients and a higher NLR indicated a worse prognosis in HCC patients treated with TACE. Patients with lower NLR values have better responses when treated with Adriamycin (Doxorubicin) combined with platinum, whereas Adriamycin alone is more effective on patients with higher NLR values. Additionally, TACE was more effective when Adriamycin was combined with Sorafenib. Hence, the choice of TACE chemo drugs could be secondary to the NLR test [50].

## 4. Types of TACE

### 4.1. Conventional TACE (c-TACE)

c-TACE is a traditional approach that involves injection of a chemotherapy drug emulsified in a water-in-oil emulsion (WOE) and oil-in-water emulsion (OWE) directly into the feeding artery, followed by injection of the embolic agent that stops blood flow to the tumor [51].

Although c-TACE has a long history of use and proven effectiveness in treating unresectable HCC, it has restrictions due to the precision of drug delivery and variable response rates [52].

According to the literature, a recent randomized controlled trial concluded that even causing more severe damage to the liver, c-TACE has lower local effects compared to DEB-TACE [51]. Whereas a meta-analysis that included six randomized controlled trials showed no difference between the two treatment modalities [53].

WOE has been demonstrated to have more embolic effects compared to OWE [48].

Once a certain amount is pooled, some of the oil overflows into the portal veins through the tumor’s drainage route. Additionally, iodized oil can enter the portal veins via the peribiliary plexus in the surrounding liver. This process can temporarily block peritumoral portal blood flow, including reversed portal blood flow into the tumor, due to the oil’s viscosity [48].

Moreover, iodized oil can pass through arterial communications, reaching neighboring hepatic arterial branches and/or extrahepatic arteries [54,55,56]. This enables embolization or identification of occult tumor feeders and may prevent the development of arterial collateral supply to the tumor following TACE [55]. Adding arterial blockage with gelatin sponge particles after iodized oil injection allows for embolization of both the hepatic artery and portal vein surrounding the tumor [48].

This approach not only causes ischemic necrosis of the hypervascular tumor portions but also induces necrosis in peritumoral areas, including tumor portions with a dual blood supply, leading to shrinkage of the surrounding liver parenchyma [55,57]. However, this also indicates that c-TACE can damage normal liver tissue, making selective catheterization essential to minimize liver toxicity. Moreover, the dose of iodized oil should be decreased as much as possible: Western countries indicate 15 mL as the maximum dose, whereas in Japan it is 10 mL [40,48].

**Figure 2:**
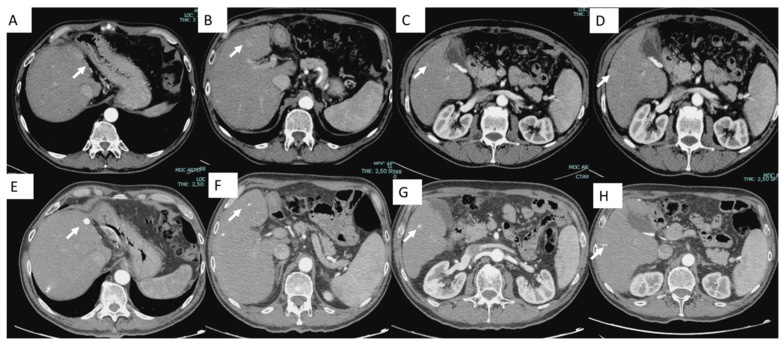
(**A**–**D**) Seventy y.o. female with multiple HCC lesions (white arrows), as shown on pre-procedural CECT treated with c-TACE. (**E**–**H**) Post-procedural CECT demonstrates the accumulation of Lipiodol in target lesions (white arrows).

From a technical perspective, chemoembolization should be as selective as possible. Selective or super-selective delivery of the chemotherapeutic agent combined with occluding particles ensures a high local concentration of the drug within the tumor and minimizes its systemic distribution. The embolic particles block the blood vessels, retaining the chemotherapeutic agent in the tumor and inducing hypoxia, which further enhances the effectiveness of the chemotherapy [43,55] (Figure 2).

A notable advantage of c-TACE is the ability to visualize Lipiodol deposition on post-procedural CT scans due to its natural radiopacity. This characteristic helps to confirm the embolized area [51]. Additionally, the radiopacity of Lipiodol facilitates real-time observation during the procedure, allowing verification of its distribution in the vascular tumor feeders [40].

Before administering Lipiodol transarterially, it is critical to assess angiography for any hepatic arteriovenous shunts. Lipiodol can traverse hepatic sinusoids and veins, potentially reaching and lodging in peripheral pulmonary arteries. While small amounts are usually not clinically significant, excessive amounts may result in symptomatic pulmonary oil embolism. The risk is especially pronounced when a shunt exists between the tumor vessel and hepatic vein, which may lead to pulmonary or systemic embolism without the operator’s knowledge [18,40].

In terms of c-TACE’s effects on the biliary system, there is an elevated risk of hepatic abscess formation if a biliary stent crosses the Ampulla of Vater or if a biliary-enteric anastomosis is present. Furthermore, the likelihood of biloma development or bile duct injury increases in cases of biliary obstruction [40].

For c-TACE, a W/O emulsion (WOE) is prepared by mixing a chemotherapy drug in aqueous solution (e.g., Doxorubicin or Epirubicin) with Lipiodol, typically in a 1:2 ratio [58]. The emulsion must be prepared immediately before injection and administered shortly after preparation, with re-homogenization possible during the procedure. This mix is created using a three-way stopcock and requires at least 20 pumping exchanges to ensure proper emulsification. To form the WOE, the syringe containing the aqueous drug solution should first be pushed into the syringe holding Lipiodol [40]. Non-ionic contrast medium can be used during the preparation of the drug aqueous solution. The rationale of this process is related to the major density that the solution reaches, increasing the stability of the WOE emulsion and decreasing the sedimentation [59].

During the treatment, Lipiodol acts as a carrier to deliver chemotherapy directly to the tumor and simultaneously causes embolization of the tumor’s microcirculation.

If the vessel is not occluded after the injection of the standard dosage, gelatin sponge particles can be used for additional temporary embolization [40]. Resorbable embolization allows a flow recanalization in 1–2 weeks and should guarantee major preservation of future patency of tumor feeders.

Peri-procedural medication should include hydration, analgesics, and antiemetic drugs. Gastric protection and antibiotics can be administered, according to the physician’s decision [40].

However, no consensus still exists regarding drugs and protocols. For example, a European randomized controlled trial performed c-TACE at a fixed schedule at 0, 2, and 6 months with the dosage of Doxorubicin calibrated based on bilirubin levels, whereas an Asian randomized controlled trial did not use a fixed scheduled for retreatment and used Cisplatin as the chemo drug [24,60,61].

### 4.2. Balloon-Occluded Transarterial Chemoembolization (b-TACE)

Balloon-occluded transarterial chemoembolization (b-TACE) is an advanced technique designed to enhance the delivery of chemotherapy and embolizing agents directly to liver tumors. This method utilizes a specialized microcatheter with a micro-balloon at its distal end. By inflating the balloon, a temporary blockage is created in the feeder vessel supplying the target lesion, which redirects the blood flow within the treatment area, allowing for a more precise and effective therapeutic intervention. Initially employed in conventional TACE, b-TACE has more recently been adapted for use in DEM-TACE [62]. This adaptation aims to improve the distribution and retention of therapeutic agents within the tumor while minimizing systemic exposure and associated side effects. In b-TACE, the blocking of the proximal arteries effect reduces the balloon-occluded arterial stump pressure (BOASP). The drug-embolic mixture (Lipiodol^®^-based or beads) can then be forcefully pushed into the tumor arteries and more intensively delivered, also filling the arterioportal micro-anastomoses at the periphery of the tumor [63].

Irie et al. demonstrated a dense Lipiodol emulsion (LE) accumulation in HCC nodules in selective balloon-occluded TACE when the balloon-occluded arterial stump pressure (BOASP) was less than 64 mmHg [64]. Under these conditions, two relatively discrete vascular compartments are created, and embolic delivery may result in reduction or elevation of the blood pressure in the downstream vascular flow maintained by anastomotic arteries such as the peribiliary plexus, interlobar communicating arcade, and isolated artery. When the blood pressure at the distal end of the catheter is lower than that of the systemic circulation, blood flowing through the arterial connections between the compartments may reverse flow into the downstream vascular compartment. This results in protection from antegrade embolization of non-target downstream arteries, in addition to the expected protection from retrograde embolization of nontarget upstream arteries [65].

One of the critical aspects of b-TACE is obtaining a detailed mapping of the arterial vasculature tumor supply to facilitate the identification of the feeders’ number and origin, ensuring precise microcatheter placement. The microcatheter should ideally be positioned in a straight vascular segment, proximal to all target lesions [66]. This strategic placement maximizes the therapeutic effectiveness of b-TACE while minimizing the risk of collateral damage. The presence of competitive feeders that cannot be occluded may affect the flow redistribution reducing the efficacy of this specific technique.

A pivotal role in the technical success of this technique is the accurate assessment of the arterial feeder size and determining the appropriate balloon inflation volume. The balloon can be inflated to diameters of 2, 3, and 4 mm, inflating predetermined fluid volumes, adhering to the manufacturer’s specified volumes and accounting for the approximate 20 s latency time to avoid overfilling or balloon rupture. Over-inflation in small vessels risks damaging the vessel wall [66], while in vessels larger than 4 mm, the balloon may not contact the arterial walls effectively, preventing the necessary pressure drop for flow redistribution. Slow and controlled balloon inflation is recommended, using a mixture of contrast media and saline. Invasive arterial pressure monitoring is recommended to evaluate BOASP. Initial pressure measurements should be in line with the targeted artery segment’s expected values. Unusually high invasive pressure may indicate incorrect catheter positioning or air bubbles in the system. Continuous monitoring during inflation will signal when the micro-balloon makes contact with the vessel walls, marked by a reduction in the pressure curve’s phasicity and amplitude. The presence of significant competitive feeders may delay or prevent the previously described pressure drop. This phenomenon is especially true in I, IV, and VIII segments, where BOASP is significantly greater compared to other segments and b-TACE advantages may be less effective [66]. Fluoroscopic monitoring provides visual confirmation of correct micro-balloon inflation and microcatheter tip positioning. Once the balloon is inflated, flow redistribution can be evaluated using standard angiographies or CBCT.

The balloon microcatheter also serves a mechanical anti-reflux function when used in collateral vessels, safeguarding extrahepatic territories (e.g., cystic, gastro-duodenal artery) or excluding arterio-portal fistulas, acting similarly to other existing fluid-controlling microcatheters [67].

Recommended chemotherapy protocols for b-TACE include Doxorubicin or Cisplatin, and the use of Miriplatin is also reported [68,69].

Chu. et al. compared the safety and efficacy of b-TACE and c-TACE in treating single HCC, with size-based subgroup analysis showing that time to LTP was significantly longer in patients with medium- to large-sized HCC (>3 cm) who underwent b-TACE rather than c-TACE, suggesting that b-TACE should be considered as an alternative option in patients with medium- to large-sized single HCC (>3 cm) who have a nonresectable condition or prefer nonsurgical treatment [70].

Lucatelli et al. compared oncological results and the safety profile of b-TACE and DEM-TACE in patients with hepatocellular carcinoma (HCC), emphasizing the role of ballon-occluded procedures as an upgrade of interventional oncology liver embolization procedures and promoting a better local tumor response, especially for medium- to large-sized single HCCs (>3 cm), with a similar adverse events rate [71].

Lucatelli et al. also evaluated the role of balloon microcatheters by comparing b-DEM-TACE to DEM-TACE and b-SIRT (selective internal radiation therapy) to standard SIRT. Balloon microcatheter-treated groups had significantly improved contrast, higher signal-to-noise ratios, and superior contrast-to-noise ratios than their conventional counterparts. Specifically, in DEM-TACE treatment, the balloon microcatheter offered a more targeted distribution of the chemotherapeutic chemicals; moreover, in this group was described a trend toward better oncological responses and longer intervals before the need for retreatment in the b-DEM-TACE group compared to DEM-TACE. This trend was particularly notable in patients with larger tumors [72]. In the SIRT groups, the use of balloon microcatheters resulted in a greater activity intensity peak indicating a more concentrated supply of radioactive particles to the tumor site. Importantly, the adverse event rates were similar between the two groups, suggesting that the use of balloon microcatheters did not significantly increase the risk of complications [73].

Irie et al. compared the efficacy of b-TACE with conventional super-selective c-TACE, demonstrating that balloon microcatheters improved the therapeutic effect and control rates of the primary nodule. However, there were no statistically significant differences in overall survival or tumor-free rates in the liver, indicating that while b-TACE may enhance local control and treatment efficacy, it does not necessarily translate to improved long-term survival [74].

### 4.3. Degradable Starch Microsphere (DSM) Transarterial Chemoembolization (DSM-TACE)

DSM-TACE is a technique based on the use of a degradable temporary embolic agent. The main object of this technique is to preserve the arterial liver supply while reducing the systemic chemotherapy dose. This treatment is, for this reason, especially suitable for multiple HCC nodules and could be administered in a lobar fashion [37].

DSMs are biocompatible amilomers, usually derived from hydrolyzed potato starch, cross-linked, and substituted with glycerol ether groups to better control the degradation rate. The degradation process of DSM primarily involves enzymatic hydrolysis by alpha-amylase, an enzyme abundantly present in human tissues. The DSM half-life is usually 40 min depending on body temperature, pH, and amylase serum levels. Commercially available DSM (e.g., EmboCept^®^ S, PharmaCept, Berlin, Germany) consists of 450 mg Amilomer, DSM 35/50, and 7.5 mL sodium chloride (60 mg/mL). DSM particles have a mean diameter of 50 μm with 75% of microspheres ranging in diameter from 20 to 200 μm.

In vivo animal studies evaluated the use of DSM infusion in the mesenteric artery, demonstrating a sharp blood flow reduction and subsequently temporary intestinal ischemia. However, both the mesenteric artery and little arterioles of the intestinal mucosa and muscularis after 25–40 min showed complete flow restoration or were sufficiently high to make intestinal ischemic damage unlikely [75,76].

Wiggermann et al. in 2013 [77] offered a comprehensive evaluation of the dynamic changes in microcirculation within hepatocellular carcinoma (HCC) lesions during the process of DSM-TACE utilizing CEUS. This study evidenced a marked decline in the parameters of peak intensity, regional blood volume, and regional blood flow when compared to measurements taken before the embolization, while gradual revascularization of the lesions was noted, as perfusion parameters approached pre-embolization levels by 90 min post-procedure [77].

Short ischemia time is thought to be responsible for reducing the response of vascular endothelial growth factor (VEGF) production after TACE. In HCC patients, VEGF expression has been correlated with aggressive behavior, early metastasis spread, vascular invasion, and poor prognosis; in patients undergoing TACE, high serum VEGF levels are predictive of poor tumor response. c-TACE appeared to be associated with a higher VEGF response in the 28 post-procedural days compared to other embolic agents, while hypoxia stimuli after DSM-TACE procedures are not strong enough to produce a VEGF response [78].

DSM-TACE, like other TACE procedures, is performed using either a transfemoral or transradial approach, requiring a detailed analysis of hepatic and lesion vascularization. Once the microcatheter is positioned correctly, the DSM infusion should be conducted under fluoroscopic guidance to minimize the risk of reflux. Current administration protocols recommend using 4 mL from a 7.5 mL DSM vial, combined with a chemotherapeutic agent to form a suspension, along with an additional 15–20 mL of contrast media. The remaining 3 mL of DSM can then be administered to further encourage vascular stasis.

**Figure 3:**
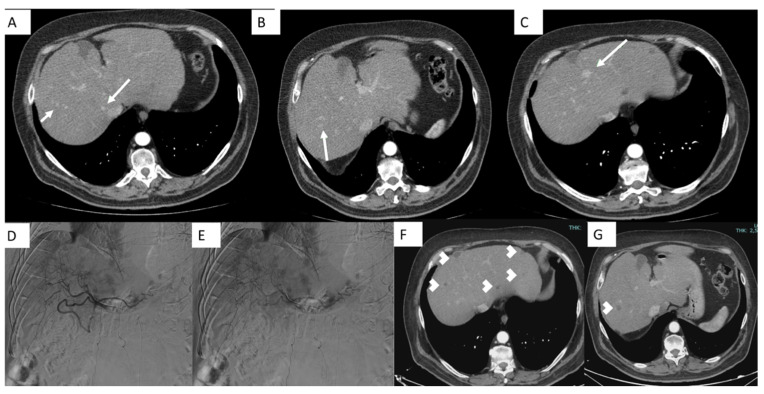
Fifty-six-year-old male affected by multifocal bilobar HCC nodules, as demonstrated on pre-procedural CECT scan (**A**–**C**) (arrows) and on intra-procedural angiograms (**D**,**E**). Four sessions (2 each lobe) of DSM-TACE were performed every 2 weeks. (**F**,**G**) Post-procedural CECT demonstrated complete response (CR) (arrowheads) and patient was candidated to liver transplantation.

The primary goals of this treatment are to deliver the full planned dose of the chemotherapeutic agent and to achieve vascular stasis. The procedure is typically repeated at least twice at each treatment site to ensure adequate coverage and therapeutic effectiveness. In cases where tumors are spread bilaterally, it is recommended to treat the lobe with the larger tumor burden first. After 14 days, treatment can proceed for the contralateral lobe. This phased approach not only ensures efficient drug absorption in the targeted lesions but also safeguards liver function by avoiding simultaneous treatment of the entire liver [37] (Figure 3).

In patients with intermediate-stage HCC and Child–Pugh score 8–9, life expectancy may be determined by liver dysfunction; the spectrum of locoregional therapies can worsen and exacerbate liver dysfunction and hurt survival. One of the main advantages of DSM-TACE is its high safety profile and its role in BCLC-A–D patients ineligible for c-TACE, SIRT, or systemic treatment. Haubald et al. in their retrospective study obtained an overall disease control rate (ODC) of 78.6% and overall median survival of 682 days. Even though the study population was composed of high-risk patients (portal vein thrombosis, extrahepatic metastases, Child–Pugh C class, BCLC-C stage, deteriorated liver function with a bilirubin level between 2 and 3 mg/dL), no post-procedural major complications were reported [79].

In retrospective studies, DSM-TACE has shown an advantage in terms of radiological and biological response over DEB-TACE (27.6 vs. 0.0%), while no benefit was found in terms of local recurrence and mortality during follow-up [80].

However, DSM advantages are not relegated to solely palliative settings; a few studies explored their role in TACE with curative intent. In a large retrospective European multicenter study, in a percentage of HCC patients, lesions could be downstaged and patients were successfully transplanted [15]. Similar results were demonstrated in single-center studies where DSM-TACE was performed as a bridging therapy [81].

Orlacchio et al. [82] retrospectively evaluated the safety and efficacy of 267 DSM-TACE procedures in 137 HCC patients (BCLC stages A, B, and C). They reported major complications in 6.8% of cases. Post-embolization syndrome was common (73.7%). According to mRECIST criteria, a high objective response rate was obtained after one treatment (84.3% of patients showed complete or partial response). The median time to progression and overall survival were 12 months and 36 months, respectively.

In another study by Orlacchio et al. [83], 24 HCC patients were prospectively enrolled to be treated with repeated DSM-TACE procedures, performed at 4–6 week intervals. Clinical and biochemical evaluations were performed before and after each procedure. No procedure-related death was observed. Complete response (CR) was observed in 5/24 (20.8%), 4/17 (23.5%), and 5/12 (41.6%) patients after the first, second, and third procedure, respectively. At the end of each treatment, all patients experienced at least a partial response.

Although some preliminary studies have investigated the differences in clinical efficacy between DSM-TACE and other more widely used types of TACE with encouraging results [80], it is common clinical practice to reserve DSM-TACE in cases where there are contraindications to performing other types of TACE given its safety profile (e.g., portal thrombosis, high bilirubin levels, etc.). Further and more in-depth studies are needed to clarify the real limitations and advantages of this technique.

### 4.4. Drug Eluting Microspheres Transarterial Chemoembolization (DEM-TACE)

DEM-TACE, first introduced by Hong et al. in 2006 [84], represents a variation of traditional TACE that employs microspheres to simultaneously deliver chemotherapy drugs and embolize tumor-feeding vessels. These microspheres function as embolic agents capable of binding drugs such as Doxorubicin, Epirubicin, Idarubicin, and Irinotecan, via ionic interactions between the cationic drug molecules and the anionic functional groups of the microspheres [85].

There are various types of drug-eluting microspheres (DEMs), each compatible with different chemotherapeutic agents, resulting in slight variations in clinical outcomes. Research has indicated that using a lower drug dosage in combination with DEMs of specific size ranges can enhance both the efficacy and safety of the treatment [86,87].

The procedure begins by loading the selected DEMs with the chemotherapy agent. Each vial is preloaded with 50–75 mg of Doxorubicin or Epirubicin, a process that typically takes 30–60 min. It is essential to prepare each DEM type according to the manufacturer’s instructions for use (IFU) [85].

Bead size has been a topic of significant discussion in recent years. Smaller beads, particularly those under 100 microns, are associated with a higher risk of hepatobiliary complications if super-selective catheterization of feeder vessels is not achieved. Recently, combining beads of different sizes has become a common practice. This approach aims to balance the deeper penetration achieved by smaller beads with the embolization effectiveness of larger ones [88].

Four permanent DEMs are approved for DEM-TACE, including DC (Drug Capable) Bead^®^, HepaSphere^®^, Embozene TANDEM^®^, and LifePearl^®^.

As for bead choice, multiple options exist, each with distinct characteristics and advantages.

**DC Beads**

DC Beads™ (BTG International, London, UK) are biocompatible, hydrophilic, non-resorbable hydrogel microspheres with precise calibration. Made from polyvinyl alcohol modified with sulfonate groups, these microspheres form a hydrogel with a high water content (95%) and are tinted blue. Typically, DC Beads are loaded with chemotherapy agents such as doxorubicin or irinotecan. The beads exhibit a high drug-loading efficiency (99%), with a maximum capacity of approximately 45 mg/mL of hydrated beads, irrespective of bead size [85].

**DC Bead LUMI**

DC Bead LUMI™ (BTG International, London, UK) are radiopaque hydrogel beads that are also biocompatible and non-resorbable. Like the conventional DC Bead, these are composed of polyvinyl alcohol but are enhanced with a tri-iodobenzyl radiopaque moiety covalently bonded to the structure. This modification makes DC Bead LUMI inherently radiopaque, enabling excellent visibility under imaging modalities such as CT, cone-beam computed tomography, and fluoroscopy. They are available in size ranges of 70–150 µm, 100–300 µm, and 300–500 µm [89].

**HepaSphere**

HepaSphere (Merit Medical, Rockland, MA, USA) microspheres are non-resorbable, biocompatible, expandable, and drug-loadable. Produced in a dry state, they are composed of vinyl acetate and methyl acrylate monomers, which form a sodium acrylate alcohol copolymer. These beads absorb surrounding fluids in aqueous environments, causing them to swell significantly while maintaining softness and deformability. This flexibility allows for delivery through most microcatheters and adaptation to vessel walls. HepaSphere beads are unique in their ability to compress up to 80% of their volume. In their dry state, they are available in sizes ranging from 30–60 µm, 50–100 µm, 100–150 µm, and 150–200 µm. While Doxorubicin is the primary drug used with HepaSphere, other agents such as Epirubicin, Irinotecan, and Oxaliplatin have also been utilized [90].

**TANDEM Microspheres**

Embozene TANDEM microspheres (CeloNova Biosciences/Boston Scientifc, Marlborough, MA, USA) are non-resorbable microspheres made of polymethacrylate hydrogel. These microspheres feature a biocompatible outer shell coated with Polyzene-F, an inorganic perfluorinated polymer applied as a nanometer-thin layer. TANDEM microspheres are capable of loading various chemotherapy drugs, including Doxorubicin-HCl, Idarubicin-HCl, Epirubicin-HCl, and Irinotecan-HCl, with a loading capacity of up to 50 mg/mL [91].

Differently from other DEBs, Embozene TANDEM typically maintains its size after drug loading to allow reliable performance and it is designed to penetrate into medium/large (35–80 μm) arterioles before blocking blood flow and releasing the drug at controlled rates, offering the smallest microspheres and the tightest calibration on the market [85].

After selecting and preparing the appropriate drug-eluting microspheres (DEMs), the operator must achieve super-selective catheterization of the tumor-feeding vessel using a microcatheter. This positioning should be confirmed through angiography or cone-beam CT (CBCT). The prepared DEMs are then mixed with a contrast agent to enhance visibility and are ready for infusion. To prevent sedimentation in the syringe, it is crucial to frequently rotate the syringe or use a three-way stopcock to gently suspend the beads in the solution.

The infusion process generally uses 1 mL or 3 mL microcatheter syringes, depending on the operator’s preference. Slow, smooth pulses synchronized with the normal arterial flow are employed to guide the beads into the tumor-feeding vessels. The infusion should be performed under continuous fluoroscopic monitoring to detect any backflow of the particles. Administration continues until vascular flow stagnates, guided by counting 10 cardiac beats [37].

Once flow stagnation is observed, the injection should be stopped, regardless of the volume of beads administered [92]. As TACE relies on both cytotoxic and anoxic effects, the goal of the treatment is complete devascularization of the tumor. This endpoint may be achieved with a small volume of beads or may require additional sessions if devascularization is incomplete, provided that the total dose of Doxorubicin does not exceed 150 mg.

Compared to other TACE techniques, DEM-TACE offers greater control over the delivered dose, reducing systemic toxicity while maintaining a high local concentration of the chemotherapeutic agent. The microspheres also allow for gradual drug release, sustaining therapeutic drug levels over a prolonged period. Furthermore, DEMs conform to and adhere to the target blood vessels, enhancing their embolization efficacy [93].

Over the years, the benefits of DEM-TACE have been well documented in both in vitro and in vivo models [93]. However, multiple studies have shown no statistically significant improvements in key outcomes such as patient survival rates and therapy response rates when comparing DEM-TACE to other methods [94,95]. Specifically, the potential superiority of DEM-TACE over traditional c-TACE has been the subject of various investigations, many of which report no substantial differences between the two approaches [87].

For instance, Brown et al. [86] conducted a randomized clinical trial at a single tertiary referral center, including 101 HCC patients who were randomly assigned to either DEB-TACE (50 patients) or transarterial embolization (TAE) with microspheres (51 patients). The study’s primary endpoint was the tumor response as assessed by RECIST 1.0, measured 2 to 3 weeks after treatment and subsequently at quarterly intervals. The evaluation was conducted by reviewers blinded to the treatment type. The results showed similar adverse event rates in both groups, and no significant differences in RECIST-based response outcomes were observed.

Similarly, a recent meta-analysis by Wang et al. compared the efficacy of DEM-TACE and c-TACE, finding that their therapeutic effects were largely comparable, with no notable differences in major complication rates [53]. That said, other meta-analyses have presented contrasting findings, suggesting that DEM-TACE might offer superior outcomes, particularly in terms of overall survival (OS) [96,97].

In summary, while recent research has questioned some of its advantages, DEM-TACE continues to be a safe, effective, and viable option for the treatment of liver tumors.

### 4.5. Hepatic Arterial Infusion Chemotherapy (HAIC)

Hepatic arterial infusion chemotherapy (HAIC) is a selective alternative to transcatheter arterial infusion (TAI) [98]. Like systemic chemotherapy, HAIC protocols require multiple cycles. While there are variations in HAIC procedures, all involve the selective catheterization of the primary hepatic artery supplying the tumor and the direct injection of the chosen chemotherapy agent [99].

The rationale for HAIC aligns with that of TACE: localized delivery increases drug concentration at the tumor site while reducing systemic exposure, thereby enhancing the anticancer effect and minimizing systemic adverse reactions [100]. For each treatment cycle, the patient undergoes a complete angiographic procedure. A catheter or microcatheter is positioned in the primary hepatic artery feeding the tumor, confirmed via angiography or CBCT. The catheter is removed after the session, and subsequent cycles involve repeated catheterization. Alternatively, a port catheter system can be implanted, akin to a venous port-a-cath, enabling less invasive procedures for drug administration. The system includes a catheter and a port, allowing repeated drug injections without requiring new catheter placements [101].

To prevent chemotherapeutic drugs from spreading to nearby organs like the stomach or duodenum, vessels such as the gastroduodenal artery or right gastric artery can be occluded using techniques like coil embolization [102].

HAIC is primarily used in Asian countries, particularly Japan, where studies have reported high response rates, favorable long-term outcomes, and manageable toxicity profiles [103]. However, no consensus has been reached as to its place as a standard treatment for advanced HCC. The principal reason is the absence of evidence of efficacy from RCTs, whether against a placebo or against an established active control treatment. The majority of RCTs on HAIC compare one unproven regimen with another unproven regimen, which primarily provides information about the toxicity but not about the efficacy of specific treatment combinations [104]. These agents were predominantly employed due to the unavailability of alternative therapeutic options and the observation that intra-arterial infusion of chemotherapeutic agents appeared to be less toxic than systemic intravenous administration. With the advent of Sorafenib as the standard of care for advanced-stage HCC, it became evident that HAIC would not offer any efficacy benefit in comparison to Sorafenib [105]. Regarding chemotherapy regimens, Cisplatin (CDDP), 5-FU plus CDDP, and 5-FU plus Interferon are among the most commonly used in HAIC, showing promising response rates and outcomes for advanced HCC [106,107,108,109]. Although Sorafenib remains the recommended first-line therapy in most guidelines [40], HAIC is often utilized in Japan for advanced HCC patients. Recent trials have investigated Sorafenib combined with HAIC, such as Sorafenib plus HAIC with Cisplatin (HAIC[CDDP]) or with 5-FU and Cisplatin (HAIC[CDDP + 5-FU]), revealing improved survival outcomes in some cases compared to Sorafenib alone [110,111]. Similarly, Lenvatinib combined with HAIC has shown promise in terms of tumor shrinkage and cost effectiveness [99].

Despite these developments, guidelines like the BCLC, AASLD, EASL, ESMO, and APASL recommend TACE over HAIC for HCC [9,98]. However, HAIC has proven effective for specific subgroups, such as patients with portal vein tumor thrombosis (PVTT). For these patients, low-dose Cisplatin and 5-FU HAIC regimens are particularly useful, as PVTT is a contraindication for TACE and often necessitates systemic therapy or palliative care [112].

HAIC has also demonstrated better outcomes in certain scenarios. For example, in patients with large (>7 cm) or huge (>10 cm) tumors without vascular invasion or metastasis, HAIC has shown superior local tumor control compared to TACE. The FOLFOX-HAIC protocol significantly improved overall survival in unresectable large HCC compared to TACE [113]. Additionally, combining HAIC with immune checkpoint inhibitors (ICIs) has shown superior responses in PVTT cases, with reduced tumor progression risk [114]. Compared to TACE, HAIC has improved overall response rate (ORR), disease control rate (DCR), and overall survival (OS) in unresectable HCC while offering a better safety profile [115].

In conclusion, while HAIC is not yet a standard treatment in most guidelines, it remains a valuable option for specific cases. Emerging protocols and combinations with systemic therapies may enhance its role in the future, potentially offering an effective alternative to systemic therapy alone.

Different studies evaluated the efficacy of different types of TACEs and compared the techniques (Table 3).

## 5. Complications

TACE-related complications are quite rare, their incidence is lower than 5%, even if some patient and/or procedure-related factors can have an impact on the incidence. Indeed, portal vein obstruction, impaired liver functional reserve, biliary obstruction, previous biliary surgery, excessive administration of embolic agent, and nonselective embolization can increase the complication rate [116].

Complications can be classified as early—either intra-procedural or post-procedural—or late.

### 5.1. Early Complications

#### 5.1.1. Intraprocedural Complications

Intraprocedural complications can be related to the puncture site or to the catheterism, such as any other kind of intra-arterial treatment. The preferred access for TACE is the femoral artery even if the radial approach is becoming more standardized [117].

The complications related to the site of puncture are mainly groin hematoma and pseudoaneurysm. Usually, hematoma is a self-limited complication, but uncontrolled hematoma or pseudoaneurysm require interventional or surgical treatment (thrombin injection, surgical evacuation, arterial suturing, covered stents) [118]. The use of US-guidance is suggested to identify the precise site of the puncture, to obtain a mono-parietal puncture, and to avoid arterial plaques or branches. All of these precautions significantly limit the incidence rate of complications. Also, using a 5 French introducer or even smaller is helpful in reducing the complication rate [117].

The complications related to catheterism are mainly spasm of the hepatic artery and arterial dissection. Arterial spasms can be elicited either by a mechanical trigger (passage of guide and catheters) or by a chemical one (chemotherapeutic and embolic drugs) [119]. Selective intra-arterial infusion of nitroglycerine might be helpful.

Arterial dissection is uncommon, but the incidence increases in patients with atherosclerosis. Dissection does not require any treatment as long as the vascular flow is preserved; in case of flow limitation, ballooning or stenting of the artery is necessary [117].

Intra-arterial thrombosis and arteritis are uncommon complications. Intra-arterial thrombosis can be secondary to spasm and dissection and it can be easily managed by heparin injection. Arteritis is characterized by arterial wall damage, caused by acute toxicity due to chemotherapeutic drugs. Acute damage eventually evolves into complete obstruction of the hepatic artery, making other intra-arterial treatments impossible. Thus, it is important to prevent this complication by using microcatheters to inject chemotherapeutic drugs [120].

#### 5.1.2. Post-Procedural Complications

Post-procedural complications are due to non-target embolization, which most commonly affects the gastrointestinal system, the spleen, the lungs, the spinal cord, the gallbladder, the pancreas, and the skin.

Nontarget embolization of the stomach or the duodenum is due to the reflux of embolic agents or the presence of undetected anatomic variants. Common anatomic variants to take into account when looking at the angiographic images are the accessory left gastric artery arising from the right hepatic artery and the right gastric artery arising from the common hepatic artery. When identified, the most common preventive measure is to avoid reflux embolization. Clinical manifestations are gastritis or duodenitis, ulceration, and bleeding [121].

Accidental embolization of the splenic artery is extremely uncommon, yet it can result in a splenic infarction or abscess. Factors that may elevate the risk of backflow include celiac stenosis paired with hepatofugal blood flow in the common hepatic artery, enhanced blood flow through the splenic artery due to liver cirrhosis and splenomegaly, and spasms of the proper hepatic artery induced by catheter use. Employing selective embolization with a microcatheter can mitigate the risk of iatrogenic complications. The primary clinical symptom is acute pain in the left upper quadrant of the abdomen, typically resolving within 3 to 10 days. In rare instances of large splenic abscesses, percutaneous drainage may be necessary [122].

Non-selective lung embolization is mostly due to an undetected intra-tumoral arteriovenous shunt. This complication is quite uncommon (incidence of 0.05%), but it can be fatal since it can induce pulmonary infarction and acute respiratory distress syndrome. The incidence increases as the dose of Lipiodol (in the case of conventional TACE) increases. Indeed, a maximum safe dose of Lipiodol has been identified, ranging from 14.5 to 20 mL [122]. The symptoms are those typical of pulmonary embolism and include cough, dyspnea, hemoptysis, and oxygen desaturation, which usually arise 1–2 days after the procedure. The gold standard to detect this condition is a chest CT-scan, which can show the typical finding of lipiodol accumulation in the lung.

A rare but serious complication is spinal cord injury. This complication is most often seen when the tumor is fed by parasitic vessels, particularly intercostal arteries. The T10, T9, and T11 intercostal arteries are commonly implicated in advanced HCC or after several TACE sessions [123].

During intercostal artery interventions, the risk of spinal cord injury exists due to the origin of spinal cord arteries from the proximal segments of intercostal arteries. The spinal cord receives its blood supply mainly from one anterior spinal artery and two posterior spinal arteries, with additional supply from radicular arteries branching from cervical, intercostal, and lumbar arteries. Despite the network of anastomoses in the spinal cord’s blood supply, embolic materials can lead to significant complications due to the anatomical and neurological layout. Collateral blood supply from intercostal arteries develops in advanced HCC or after multiple TACE sessions, raising the risk of unintended embolization of spinal branches and resulting in paraplegia [123].

Non-target embolization of the cystic artery or accessory cystic artery can result in acute cholecystitis, presenting with typical symptoms such as fever and abdominal pain. This complication is relatively common, occurring in 0.3% to 10% of cases [124]. When cholecystitis is caused by an infarcted gallbladder, cholecystectomy is often required to prevent progression to gallbladder necrosis. Prompt surgical intervention usually leads to a favorable outcome.

#### 5.1.3. Acute Pancreatitis

Acute pancreatitis, though rare (0.9–2%), can occur as a result of reflux embolization into the dorsal pancreatic or gastroduodenal artery. The gastroduodenal artery is a terminal branch of the common hepatic artery and supplies the pancreatic head and uncinate process, which are especially susceptible to reflux embolization. Symptoms include abdominal pain, fever, vomiting, and elevated levels of amylase and lipase. Conservative management is usually sufficient, but when pancreatic necrosis is suspected on CT imaging, antibiotics may be necessary to prevent bacterial translocation [125].

#### 5.1.4. Skin Injury

Non-target embolization of cutaneous branches, such as the hepatic falciform artery, can lead to skin injury in 2–24.5% of angiographic cases. The hepatic falciform artery, which originates from the left or middle hepatic artery, traverses the falciform ligament to supply the area around the umbilicus, occasionally causing an epigastric skin rash after TACE. Treatment typically involves oral nonsteroidal anti-inflammatory drugs, with the skin lesions generally resolving within a year [126].

### 5.2. Late Complications

#### 5.2.1. Liver Failure

A temporary decline in liver function is common after TACE, but irreversible liver failure can occur, characterized by an increase in serum bilirubin, worsening ascites, or the development of hepatic encephalopathy within two weeks of the procedure. This severe complication increases both morbidity and mortality. Portal vein thrombosis is a major risk factor, as it reduces liver perfusion. Despite advances in microcatheter technology, which allow for TACE even in cases of main portal vein thrombosis, liver transplantation remains the definitive treatment for some patients. Supportive care, including IV hydration, pressure support, and management of hepatic encephalopathy, is usually provided [127].

#### 5.2.2. Renal Failure

Renal failure due to contrast-induced nephrotoxicity may occur, defined by a more than 50% increase in serum creatinine levels or levels exceeding 1.5 mg/dL within seven days of the procedure. Prophylactic hydration and the use of low-osmolality contrast agents are effective preventive measures to reduce the risk of renal failure [128,129].

#### 5.2.3. Liver Infarction

Hepatic infarction is an uncommon complication because of the liver’s dual blood supply from both the hepatic artery and portal vein. However, it can lead to liver abscesses or sepsis. Portal vein thrombosis is a known risk factor. Imaging typically reveals hypodense areas on CT and hypointense T1-weighted or hyperintense T2-weighted regions on MRI. Management includes prophylactic antibiotic therapy to prevent secondary infections [122,130,131].

#### 5.2.4. Liver Abscess

Liver abscesses, occurring in 0.3–1.3% of cases after TACE, are often the result of tumor necrosis, which creates an environment favorable for bacterial growth. The risk of abscess formation is higher in patients with leukopenia, immunodeficiency, diabetes, or bilio-enteric anastomoses. Some studies suggest that injecting antibiotics along with embolic agents during TACE can reduce the incidence of liver abscesses. Smaller abscesses (less than 5 cm) are usually managed conservatively, while larger ones (greater than 5 cm) may require drainage [132,133,134].

#### 5.2.5. Tumor Rupture

Tumor rupture, though rare, is a life-threatening complication with a mortality rate ranging from 25% to 75%. This is often due to reactive tissue edema caused by TACE and vasculopathy associated with malignancy. Endovascular embolization is generally the treatment of choice, although surgical or expectant management may be considered in certain cases [135,136,137].

#### 5.2.6. Biliary Complications

Biliary complications, including strictures, are observed in 0.5–10% of TACE procedures. These occur because the bile ducts are solely vascularized by the hepatic artery, making them vulnerable to necrosis, ectasia, biloma formation, or stenosis. Drug-eluting microsphere TACE (DEM-TACE) has been associated with increased bile duct damage compared to conventional TACE (c-TACE). However, patients with advanced cirrhosis may have a lower risk due to hypertrophy of the peribiliary vascular plexus, which compensates for reduced hepatic artery flow. Management includes conservative care, antibiotics, and biliary drainage as necessary [138].

#### 5.2.7. Post Embolization Syndrome (PES)

PES is not considered a (TACE) complication itself since it is the most frequent adverse event after any kind of embolic treatment. The underlying mechanism responsible for PES seems to be related to the release of inflammatory cytokines in response to ischemic tissue and to the systemic effects of the chemotherapeutic agents.

However, the incidence of PES is similar in patients undergoing TACE and in those undergoing transarterial embolization (TAE), thus the effect of chemotherapeutic drugs seems to be less involved in the pathogenesis of PES [139]. The diagnosis is based on clinical presentation that is usually characterized by the classic triad of fever, abdominal pain and leukocytosis, beginning up to 72 h after the procedure. Nausea and/or vomiting can be present. A recent retrospective observational study proposed a scoring system for the classification of PES, categorizing the syndrome into mild PES, moderate PES, and severe PES [140]. Despite the severity, symptoms are usually self-limited, and only supportive care is needed, mainly antiemetic drugs (5HT3 antagonists) and analgesics. Nonetheless, PES is related to prolonged hospital stays and increased costs for the healthcare system. For this reason, several studies aimed to identify patient- and procedure-related predictors of PES, but up to now only potential risk factors have been described, such as younger age, absence of cirrhosis, gallbladder embolization, and tumor size [141,142].

Moreover, some authors suggest that PES should be considered an independent predictor of poor outcomes in terms of overall survival following TACE [143].

There is no consensus about treatment to prevent PES. The use of Dexamethasone, either intra-arterial (together with the mixture of Lipiodol and chemotherapeutic) or following the procedure, is controversial. Some studies suggest that prophylactic administration of Dexamethasone before TACE is effective in reducing PES incidence [144], others discourage its use [139].

## 6. Follow-Up and Response to Treatment

The imaging modalities of choice to assess response to treatment after TACE (as well as for other locoregional treatments on the liver) are CT-scan and MRI. The radiological response can be evaluated with different criteria. The most recognized ones are the World Health Organization (WHO) criteria, the Response Evaluation Criteria in Solid Tumors (RECIST), or the Liver Imaging Reporting and Data System (LI-RADS) assessment score [145,146].

MRI is the preferred imaging modality according to sensitivity and specificity. Nonetheless, sometimes CT-scan is preferred due to its higher availability and in some specific cases in which MRI is not feasible (absolute contra-indications) or it is less sensitive, such as in patients with ascites. Nevertheless, comparison with the same imaging technique used pre-treatment is recommended for better diagnostic accuracy [147].

The timing of the follow-up after TACE is at 1 and 3 months and then every 3 to 6 months after treatment. The radiological response at 1 month is crucial since it has been associated directly with the overall survival rate [148].

The imaging protocol, both for CT and MRI, is characterized by a multiphasic acquisition (non-contrast acquisition, late arterial phase, portal venous phase, and delayed phase) [149].

MRI protocols should also include T2-weighted imaging both with and without fat suppression, T1-weighted in- and opposed-phase sequences, diffusion-weighted imaging (DWI), and unenhanced followed by dynamic contrast-enhanced (DCE) 3D gradient-recalled echo fat-suppressed imaging using either gadolinium-based extracellular or hepatobiliary agents with subtraction imaging [150].

After imaging acquisition, imaging evaluation should be performed always according to the same response criteria. Moreover, the presence of nontarget embolization or of other complications must be taken into account when evaluating the images.

## 7. Future Perspectives

The introduction of transarterial chemoembolization (TACE) into clinical practice marked a pivotal development in the treatment of hepatic tumors, and over the years, several variations of this technique have emerged. Alongside these innovations, there has been a notable expansion in the array of pharmaceutical agents available for use. Despite significant advancements, current research continues to explore promising new approaches, including novel TACE drugs, combinations of TACE with immunotherapy, and the use of TACE in conjunction with ablation therapies.

Lenvatinib (LEN) is widely considered a standard first-line treatment for advanced hepatocellular carcinoma (HCC); however, survival outcomes for this patient group remain suboptimal. In the LAUNCH trial [151], a randomized phase III study, the efficacy of systemic Lenvatinib alone was compared with the combination of Lenvatinib and TACE (LEN-TACE) in 338 patients across 12 medical centers. Participants were randomly assigned to receive either LEN-TACE (170 patients) or LEN alone (168 patients). After a median follow-up of 17 months, the median overall survival (OS) was significantly longer in the LEN-TACE group (17.8 months vs. 11.5 months; *p* < 0.001). Additionally, progression-free survival (PFS) in the LEN-TACE cohort was 10.6 months, compared with 6.4 months in the LEN-only group (hazard ratio, 0.43; *p* < 0.001). The objective response rate (ORR) based on modified RECIST was higher in the LEN-TACE group (54.1% vs. 25.0%, *p* < 0.001). Although hepatotoxicity of grade 3–4 occurred more frequently in the LEN-TACE group, the rates of dose reduction or interruption of LEN were similar across both groups. The study concluded that the combination of LEN with TACE is more effective than LEN monotherapy for controlling tumors and extending survival, with an acceptable side effect profile.

Blocking the interaction between PD-1 and its ligands has gained traction as a promising therapeutic strategy for HCC. Consequently, several clinical studies have investigated the potential benefits of combining TACE with anti-PD-1/anti-PD-L1 inhibitors. Zhang et al. [152] assessed the safety and efficacy of combining TACE with the immune checkpoint inhibitor (ICI) Camrelizumab in patients with unresectable HCC. Their results indicated that this combination therapy led to effective tumor control (ORR of 35.5%) and enhanced survival compared to ICI monotherapy. The ORR observed in this combination approach was superior to that reported for other ICIs, such as Nivolumab (CheckMate-040) and Pembrolizumab (KEYNOTE-240), where the ORRs were 15–20% and 18.4%, respectively [153,154]. These findings suggest that TACE combined with Camrelizumab offers a promising treatment option, though additional prospective studies are needed for further validation.

TACE has been a mainstay in the treatment of unresectable HCC (uHCC) for over two decades, yet most patients with uHCC experience disease progression within a year. The EMERALD-1 trial, a global Phase III, double-blind study [155], examined the effects of combining Durvalumab with placebo (D + TACE), Durvalumab with Bevacizumab (D + B + TACE), or placebo with placebo (TACE) following one to four TACE procedures. In total, 616 patients with BCLC stage A (25.8%), stage B (57.3%), and stage C (16.1%) were randomized to receive either D + B + TACE (*n* = 204), D + TACE (*n* = 207), or TACE alone (*n* = 205). The study found that PFS was significantly longer in the D + B + TACE group compared to TACE alone (15.0 vs. 8.2 months; hazard ratio [HR], 0.77; 95% confidence interval [CI], 0.61–0.98; *p* = 0.032). Results were consistent across various subgroups. The secondary comparison between D + TACE and TACE alone did not show statistical significance. These results demonstrate that the combination of Durvalumab and Bevacizumab with TACE improves progression-free survival compared to TACE alone, making it the first ICI-based regimen to show significant clinical improvement in PFS.

**Figure 4:**
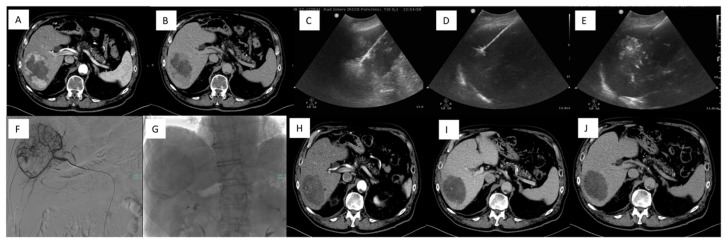
Seventy-five-year-old male affected by a residual HCC after MWA treatment of 9 cm of enhancing area with wash-out (**A**,**B**). Combined treatment with MWA with multiple antenna insertions by US-guidance (**C**–**E**) and b-TACE with HepaSphere 50–100 μm and Epirubicin, as demonstrated in angiograms (**F**,**G**). One month post procedural follow-up demonstrated complete response to treatment (**H**–**J**).

Combination therapies have also been applied effectively in the treatment of early- and intermediate-stage HCC tumors greater than 3 cm, showing superior results compared to TACE or ablation alone [156]. Achieving a curative outcome requires maximizing the volume of necrosis induced by the treatment. Early studies suggest that combining TACE with ablation enhances tumor control and the overall effectiveness of the procedure [157] (Figure 4).

Iezzi et al. [158] performed a case series to demonstrate that b-MWA plus b-TACE could be a safe and effective combined therapy for unresectable large HCC lesions, allowing a high rate of local response also in lesions exceeding 5 cm in size, obtaining a mean necrotic area of 6.8 ± 0.47 cm (range 6.3–7.4 cm).

Another future perspective is the use of inflammation-based scores, such as the neutrophil-to-lymphocyte ratio (NLR), lymphocyte-to-monocyte ratio (LMR), and platelet-to-lymphocyte ratio (PLR), as prognostic indicators in various cancers. However, their predictive role in patients with intermediate-stage HCC undergoing transcatheter arterial chemoembolization (TACE) remains an area that requires further investigation, although early recognition of TACE refractoriness holds the potential to guide tailored therapeutic interventions.

A study by Minici et al. [159] concluded that LMR and NLR can be useful in predicting the treatment response and short-term outcomes of patients with intermediate-stage HCC undergoing TACE. Future investigations should focus on validating the clinical applicability of these scores and assessing their impact on long-term patient survival and therapeutic decision making. Future investigations should focus on validating the clinical applicability of these scores and assessing their impact on long-term patient survival and therapeutic decision making.

## 8. Artificial Intelligence

The patient selection criteria of TACE and other IR treatments are nowadays based primarily on serum markers and imaging features defined by radiologists, but due to the variability in treatment response, the evaluation of different parameters of disease, including quantitative imaging features, serum markers, and functional biomarkers, is needed to improve patient management [160,161,162].

Artificial intelligence (AI) contains computing algorithms capable of performing activities often associated with human intelligence, with varying degrees of autonomy [163,164,165].

AI encompasses both machine learning (ML) and deep learning. Machine learning (ML) uses the “reverse training” method to focus on specific pathological features during training. Once trained, the computer can apply the information to new cases. Artificial neural networks (ANNs) are a commonly used machine learning techniques [166,167,168].

A convolutional neural network (CNN) is a deep artificial neural network that is ideal for imaging. Convolutional neural networks are inspired by the connectivity structure of visual cortex neurons, which scan an image with receptive fields and feed it into a deep neural network. Convolutional neural networks, like artificial neural networks, have input and output, as well as several hidden layers [169,170,171,172,173].

Radiomics is a field where artificial intelligence can be applied. Radiomics is an extension of computer-aided diagnosis that gives computer-extracted characteristics related to tumor biology as well as other clinical, pathologic, and genetic data [174,175,176,177,178,179,180].

Following image acquisition, the process of radiomics for HCC typically begins with manual or semi-automatic image segmentation of a single tumor area or the entire tumor volume [181,182,183]. Texture features are then typically extracted using dedicated radiomics software. Features can be categorized into semantic and agnostic data. Radiologists generally employ semantics to define tumors, which can include size, shape, location, vascularity, necrosis, etc. Agnostics data include histograms, haralick textures, laws textures, wavelets, Laplacian transforms, and others. The combination of agnostic and semantic data generates a large amount of data, which may be examined and integrated with clinical information to improve ML models [181,182].

Recently, the development of artificial intelligence and the application of radiomics with its digital encoding of radiological pictures into quantitative features, including size, textural patterns, and shape indicators, have played a crucial role in treatment decisions and prediction of liver and other solid tumor response and overall survival [181,184,185].

We can consider two broad modeling paradigms for applying AI to the assessment of tumor response to a specific treatment. The first paradigm involves the utilization of both pre-treatment and post-treatment data as model features to educate AI to determine whether treatment was effective or not. The second paradigm involves only data available prior to treatment for the extraction of features or model inputs to predict whether a treatment will be effective [181,186,187,188,189].

Yu et al. [190] created a logistic regression model to predict a sustained complete response to TACE using mRECIST criteria, using a targeted proteomics approach that combined five candidate marker proteins (LRG1, APCS, BCHE, C7, and FCN3) with additional clinical features such as tumor number, baseline AFP, and baseline Prothrombin induced by vitamin K absence-II (PIVKA-II). This proteomics-driven logistic regression model achieved an area under the curve (AUC) of 0.813 on a testing dataset of 80 patients after utilizing a training dataset of 100 patients.

Morshid et al. [191] analyzed the CECT scans of 105 patients to predict response based on pre-TACE imaging and clinical data. They classified HCC as TACE-susceptible or TACE-refractory based on mRECIST criteria on post-TACE imaging.

Peng et al. [192] evaluated 1687 CT image patches extracted from 562 patients prior to TACE to predict four categories of TACE treatment response at follow-up according to mRECIST (complete response, partial response, stable disease, and progressive disease).

A novel combined model consisting of a radscore of nine radiomics features and clinical characteristics (pre- and post-TACE) has been proposed by Dai et al. to predict the outcome of patients with HCC treated with TACE [193].

Advancing the integration of radiological and clinical features into a combined model is essential for enhancing patient selection and optimizing c-TACE protocols [194,195,196].

## 9. Discussion

Chemoembolization represents a cornerstone of hepatic oncological interventional practice, with a predominant role in patients with unresectable HCC.

Although it has been an established procedure for decades, TACE does not currently benefit from fully standardized protocols. For instance, according to CIRSE-endorsed guidelines [37], Gram-antibiotic prophylaxis is recommended for TACE patients with risk factors to develop liver abscesses (e.g., biliary stenting, incompetent Oddi sphincter); nevertheless, there is still an open debate about considering prophylaxis in all patients, as supported by an extensive consensus statement [18] and a recent meta-analysis, stating that the therapy reduced the occurrence of post-TACE liver abscesses by 2/3 [197]. Another dispute is the appropriate use of embolization materials after TACE. The ideal embolic agent should occlude the vessels at the precapillary level [198]. For this reason, both temporary occluders and permanent embolizing agents can give benefits, the former providing a temporary embolization, hence leaving space for eventual future treatments, and the latter, given the possibility to choose particles of appropriate size, can both preserve the patency of extra- tumoral arteries and limit the complications of inadequately small particles (e.g., pulmonary embolism or biliary ischemia) [199]. Finally, the embolization endpoint is a more complex issue to build a consensus upon because it largely depends on the grade of selectivity of the catheter cannulation (selective or super-selective) and tumor-related factors (e.g., size, vascularization) [200], so the consensus moves toward adapting every single treatment to the patient and tumor to treat. The aforementioned factors can significantly impact the efficacy and safety of the procedures. Hence, greater standardization in drawing a standard line would be crucial. Another considerable discussion in the field of treatment of locally advanced liver cancer is the argument that transarterial embolization alone (TAE) could provide similar benefits when compared to TACE. In consideration of the first high-quality historical RCT that highlighted the efficacy of TACE and because intra-arterial injection of chemotherapeutic drugs anticipated the modern version of TACE, fewer RCTs focused on TAE [201,202]. The main controversy of this dualism is because the embolic effect may override the impact of the chemotherapeutic, especially in more extensive tumors. The literature on the topic is heterogeneous, both in terms of study quality and results, with several reports stating the clinical superiority of TACE. Recently, Wang et al. published a systematic review comparing the results of six RCTs [203] enrolling 683 patients. There were no significant differences between TACE and TAE for progression-free survival (HR 0.83, 95% CI 0.45–1.55; *p*  =  0.57), overall survival (HR 1.10, 95% CI 0.90–1.35; *p*  =  0.36), and objective response rate (OR 1.17, 95% CI 0.80–1.71; *p*  =  0.42), without obvious publication bias. The predominant application of TACE may not only be attributable to more extensive literature about its results but also to the fact that higher volumes of embolized tissue are required in bland embolization procedures, potentially making repeated procedures harder [204]. Eventually, given its pivotal role in managing HCC, regrouping current knowledge and expert opinions would be of utmost importance.

The role of artificial intelligence and radiomics has been rising in the last years, with the introduction of models both for the diagnosis of HCC and for prediction of tumor response, leading to a more standardized treatment for each patient [176].

## 10. Conclusions

The role of TACE has been the subject of extensive investigation, with robust scientific evidence attesting to its safety and efficacy. Further analysis is required to determine which drug is most effective, which TACE method is most effective, and which indications are most appropriate. Nevertheless, in accordance with the tailored approach of modern medicine, the existence of diverse chemoembolization techniques does not imply a preference for one over the others. Instead, it offers a broader range of potential techniques that can be employed to combat hepatocarcinoma. The success of this approach relies on the expertise of multidisciplinary oncology teams and a thorough understanding of the latest interventional possibilities.

To date there is still no consensus in the literature about the superiority of different TACE techniques. The heterogeneity of study protocols and the follow-up timing represent two of reasons for this lack of agreement. This variability mainly depends on different types of patients selected, as the procedure is indicated as first-line treatment both for intermediate (BCLC-B) and as alternative to other not practicable or failed curative treatments (surgical resection, local ablation, systemic therapies) in very early (BCLC-0) and early (BCLC-A) patients. Another variability is represented by the number of treatments performed and type of TACE for each patient. Future randomized controlled trials are indispensable to clarify the role of different types of TACE for the treatment of HCC.

## Figures and Tables

**Table 1:** Summary of the characteristics of different types of TACE, including the types of particles, chemotherapeutic dosage, advantages, and disadvantages of different techniques.

TACE	Type of Particles	Chemotherapeutic Dosage	Advantages	Disadvantages
cTACE	Chemotherapeutic agent mixed with iodized oil (Lipiodol)	50–75 mg of Doxorubicin Max 150 mg per single treatment	-Embolization of small occult tumor feeders -Cheap -Intra- and post-procedural visualization of Lipiodol particles	-Variable response rate -Systemic symptoms usually require pre-medications
DSM-TACE	Degradable starch microsphere (Embocept)	Doxorubicin at a dose of 50 mg/m^2^ adjusted for body surface	-Administration in lobar fashion -Feasible in patients with PVT, Child–Pugh score 8–9, ineligible for other locoregional or systemic treatments -Lower liver toxicity -Temporary embolic effect	Requires at least two procedures
DEM-TACE	DC Bead, HepaSphere, TANDEM, DC Bead LUMI, etc.	50–75 mg of Doxorubicin Max 150 mg per single treatment	-Wide choice of particle types and sizes -Chemotherapeutic release sustained for a longer period -Stronger embolizing effect	-Not feasible in patient with PVT or high Child–Pugh score -Requires microsphere preparation (>40 min)
b-TACE	Could be combined with different types of particles	50–75 mg of Doxorubicin Max 150 mg per single treatment	-Anti-reflux system -Increased chemotherapeutic dosage accumulation in the nodule -Could be combined with different particles and techniques	-Arterial vessel diameter ≤ 4 mm Longer procedural time -Higher risk of vessel wall damage -High cost of the micro-balloon catheter
HAIC	No use of particles	Depends on chosen chemotherapy protocol	-PVT not a contraindication -Better outcome for HCC nodules > 5 cm	Requires multiple procedures or arterial port-a-cath implantation

**Table 2:** Protocol of Doxorubicin dose and technical consideration during c-TACE, DEM-TACE, and DSM-TACE.

**c-TACE**	Doxorubicin dose: 30–75 mg/m^2^, up to 150 mg. Mixed with 5–20 mL Lipiodol.
**DEM-TACE**	-Limited Disease: 50–75 mg Doxorubicin loaded into 2 mL DC Beads (1 vial). -Advanced Disease: Up to 150 mg Doxorubicin loaded into 2 vials.
**DSM-TACE**	-Slow injection of the first 4 mL of EmboCept (450 mg/7.5 mL) mixed with 6 mL of non-ionic contrast medium and 50 mg/m^2^ of Doxorubicin diluted in 5–10 mL of saline solution -Residual 3.5 mL of EmboCept, added with an equivalent volume of contrast medium until flow stasis is reached.

**Table 3:** Summary of studies analyzing the treatment of HCC using various TACE techniques, including randomized controlled trials, observational studies, and systematic reviews.

Reference	Type of Study	Key Results
Irie T, Kuramochi M, Takahashi N (2013) [67]	Observational study	Balloon-occluded transarterial chemoembolization (b-TACE) improved Lipiodol accumulation, enhancing tumor targeting efficacy.
Chu HH, Gwon D IL, Kim GH, et al. (2022) [72]	Observational study (propensity score)	b-TACE showed better local tumor control and fewer complications compared to conventional TACE for single hepatocellular carcinoma.
Lucatelli P, De Rubeis G, Trobiani C, et al. (2022) [75]	Retrospective cohort study	b-TACE demonstrated improved response rates compared to DEB-TACE, particularly in patients undergoing micro-balloon interventions.
Irie T, Kuramochi M, Kamoshida T, Takahashi N (2016) [77]	Observational study	b-TACE showed improved outcomes for small hepatocellular carcinoma compared to conventional TACE, with higher overall response rates.
Wiggermann P, Heibl M, Niessen C, et al. (2012) [80]	Observational study	DSM-TACE evaluated using DCE-US demonstrated good tumor response and promising efficacy for hepatocellular carcinoma treatment.
Schicho A, Hellerbrand C, Krüger K, et al. (2016) [81]	Observational study	Different embolic agents used in TACE procedures were associated with varied systemic VEGF level changes, influencing tumor angiogenesis.
Brown KT, Do RK, Gonen M, et al. (2016) [89]	Randomized controlled trial	Doxorubicin-eluting microspheres showed similar efficacy but better safety compared to bland embolization in hepatocellular carcinoma.
Kloeckner R, Weinmann A, Prinz F, et al. (2015) [90]	Observational study	Drug-eluting bead TACE (DEB-TACE) showed better local disease control compared to conventional TACE in hepatocellular carcinoma.
Lammer J, Malagari K, Vogl T, et al. (2010) [97]	Randomized controlled trial	DEB-TACE was effective in treating hepatocellular carcinoma with reduced systemic toxicity compared to conventional TACE.
Golfieri R, Giampalma E, Renzulli M, et al. (2014) [98]	Randomized controlled trial	DEB-TACE achieved comparable tumor response to conventional TACE but offered better tolerability and fewer adverse events.
Yang B, Liang J, Qu ZY, et al. (2020) [99]	Systematic review	Reviewed various transarterial strategies, concluding that DEB-TACE was safer and more effective than conventional TACE in many cases.
Liu K, Zheng X, Lu D, et al. (2023) [100]	Observational study	DEB-TACE combined with molecular targeted agents improved survival rates and treatment efficacy for unresectable hepatocellular carcinoma.
Ikeda M, Shimizu S, Sato T, et al. (2016) [103]	Randomized phase II trial	Sorafenib combined with Cisplatin infusion improved progression-free survival compared to Sorafenib alone in advanced hepatocellular carcinoma.
Kudo M, Ueshima K, Yokosuka O, et al. (2016) [113]	Randomized controlled trial	Sorafenib combined with low-dose Cisplatin infusion showed no significant survival benefit compared to Sorafenib alone but had higher treatment-related adverse events.
Wang et al., 2020 [55]	Observational Study	DEB-TACE showed advantages over conventional TACE in terms of efficacy and patient outcomes.
Orlacchio et al., 2020 [85]	Prospective Cohort Study	DSM-TACE demonstrated promising long-term results for unresectable HCC patients.
Orlacchio et al., 2018 [86]	Prospective Pilot Study	Repeated DSM-TACE was safe and effective for unresectable HCC patients.
